# Considerations in the Management of a Kidney Transplant Patient With HIV

**DOI:** 10.7759/cureus.18744

**Published:** 2021-10-13

**Authors:** James Gilbert, Ali Manji

**Affiliations:** 1 Surgery, Christ Church, University of Oxford, Oxford, GBR; 2 Medical Sciences, University of Oxford, Oxford, GBR

**Keywords:** graft rejection, immunosuppression, antiretroviral therapy, hiv associated nephropathy (hivan), renal transplant, hiv/aids

## Abstract

In the wake of highly active antiretroviral therapy (HAART), kidney transplantation has become common practice in HIV-positive recipients. However, management is more complex than that of a seronegative recipient in the pre-operative, peri-operative, and post-operative periods. Although the standard HAART regimen is often modified to improve outcomes and reduce interactions with the post-transplant immunosuppressive regimen, kidney transplantation in HIV-positive individuals is feasible, with high graft survival rates comparable to those in their seronegative counterparts. There is also increasing interest in the possibility of HIV-positive kidney donation, which could increase the donor pool in seropositive patients with end-stage renal disease. This report highlights considerations in the management of a seropositive kidney recipient, reviewing the evidence that underpins current treatment guidelines and highlighting the role of HAART in the dramatic change in attitude towards transplantation in this population. It also addresses studies from multiple countries which have shown favourable outcomes in transplants from HIV-positive donors. This warrants further investigation into seropositive-to-seropositive transplantation as a potential therapeutic option.

## Introduction

Kidney transplantation is the long-term treatment goal for patients with end-stage renal disease (ESRD), in which the estimated glomerular filtration rate drops below 15 mL/min and the patient is dependent on dialysis for the clearance of circulatory waste products [1]. Fifty-four per cent of patients requiring renal replacement therapy (RRT) receive a transplant; dialysis makes up the remainder, with 40% opting for haemodialysis and 6% peritoneal dialysis (PD) [1]. Haemodialysis requires prerequisite surgery to form a fistula, with subsequent dialysis at a local haemodialysis centre triweekly. PD catheter insertion allows patients to self-dialyse at home but requires patient training and can involve (in the case of continuous ambulatory PD) up to four sessions a day.

The evaluation of a potential kidney transplant recipient is very thorough, with strict criteria on contraindications and comorbidities to be considered. This is in part due to the lengthy waiting times before transplantation and also due to significant post-transplant complications both from the surgery and, more importantly, from the complex, life-long immunosuppressive regimen patients face afterwards [2]. Absolute contraindications to transplantation include active infections or malignancy, mental health illness with a lack of capacity, drug and alcohol addictions, kidney failure that is reversible, and significantly shortened life expectancy [2].

Relative contraindications to renal transplantation include cardiovascular disease, diabetes mellitus, infection and a history of malignancy [2]. A history of cardiovascular disease or diabetes increases the risk of perioperative or early post-operative morbidity and mortality and requires careful screening before the decision to transplant is made. Infections are another concern in ESRD patients awaiting transplant, even if they are latent or controlled. The immunosuppressive nature of post-transplant treatment carries with it a risk of reactivation and complications associated with the particular infection. Hepatitis B or C positivity requires a hepatology review pre-transplant, and HIV testing is routine for transplant candidates - as seropositivity impacts the management of a recipient [2,3]. In a similar manner, patients with a history of malignancy must meet certain eligibility criteria; they are normally considered for transplant depending on the potential for recurrence or progression, and the aim is to minimise the risk of micrometastasis development whilst immunosuppressed.

## Case presentation

A 66-year-old lady (who provided written consent for the publication of this case report) presented to her General Practitioner in 2018 with long-standing lethargy, a loss of 15 kg over two years and lower-than-normal urine output. She was diagnosed with ESRD following blood tests and renal biopsy and was referred to the dialysis access assessment clinic. She elected for PD and had her catheter inserted surgically. She briefly underwent a period of haemodialysis via a tunnelled Tesio line (in her internal jugular vein) due to an episode of PD peritonitis, before reverting to PD. She continued with PD until her transplant procedure in February 2020.

The patient has an extensive past medical history, for which she takes numerous medications including alfacalcidol (a vitamin D analogue) following the development of ESRD, and pravastatin and ramipril for cholesterol and hypertension management. She was diagnosed with Graves’ disease in 2015, but her elective thyroidectomy was cancelled as it clashed with her transplant procedure. She also carries the sickle cell trait. Most importantly with regards to her renal transplant is her history of hepatitis B and HIV, having been HIV-positive since 2003. She attends check-ups every two months, and her antiretroviral therapy (ART) consists of 600 mg abacavir and 300 mg lamivudine (both nucleoside reverse transcriptase inhibitors or NRTIs) daily. At the time of transplant assessment, her plasma viral load was undetectable. It is thought that her ESRD was secondary to HIV nephropathy and hypertension.

The patient received a right kidney from a donor after brain death (DBD), with a 1/1/1 HLA mismatch. The donor was CMV antibody negative and HIV negative. The kidney was implanted extraperitoneally into the right iliac fossa in a standard fashion via a Gibson incision. The donor renal artery and vein were anastomosed to the external iliac artery and vein, respectively. The ureter was then anastomosed to the patient’s bladder mucosa using the Lich-Gregoir technique and stented. Haemostasis and perfusion were ensured before the abdomen was closed. The cold ischaemia time was 10 hours and 11 minutes.

Following transplant, the patient was put on an intensive immunosuppressive regimen. Her induction therapy comprised basiliximab and methylprednisolone. Longer-term maintenance therapy included tacrolimus and mycophenolate mofetil. She received a conventional tacrolimus dose of 6 mg, taken orally twice a day. A number of antiretroviral drugs interact with tacrolimus, particularly protease inhibitors (PIs), and many HIV-positive patients on PI therapy receive reduced tacrolimus doses to prevent transplant nephrotoxicity [4]. However, the patient's antiretroviral regimen did not include a PI, and as such high dose tacrolimus was not contraindicated. She was also prescribed drugs for prophylaxis against common opportunistic infections; isoniazid for tuberculosis, co-trimoxazole for Pneumocystis, and valganciclovir for cytomegalovirus. Allopurinol was prescribed to prevent renal transplant-associated hyperuricemia and gout. Other post-operative drugs included cyclizine and ondansetron as anti-emetics, patient-controlled analgesia (which was stopped after three days and replaced by oral analgesics), and Dalteparin as low molecular weight heparin. The patient’s immunosuppressive regime differed slightly from the standard array of drugs, however, as she was also prescribed alemtuzumab as induction immunosuppression.

A renal ultrasound scan eight hours post-transplant showed a normal greyscale appearance, no hydronephrosis, and normal Doppler waveforms. A small amount of ascites in the left iliac fossa was attributed to PD. Intraoperative histopathology of iliac lymph nodes revealed only benign reactive hyperplasia. We examined the patient three days post-transplant. She was fatigued with a weak voice and had not yet mobilised. Her lungs were clear and her abdomen was tender only above her transplant scar. A clinical review one week post-transplant showed stable blood pressure and falling creatinine. There were no urinary or viral symptoms, and the surgical site healed by primary intention with no signs of infection. The patient was discharged shortly after for review in a transplant follow-up clinic.

## Discussion

HAART and transplant eligibility

Our patient’s successful transplant, given her HIV-positive status, is an important discussion point. HIV seropositivity was an absolute contraindication for solid organ transplant in many centres before the advent of highly active antiretroviral therapy (HAART). Even in the HAART era, where a normal life expectancy is common in treated patients, transplant eligibility is not a guarantee, with some suggestions that immunosuppression following transplant might accelerate the patient's disease progression [5]. This raises a number of important ethical questions. As transplantable organs are scarce, their allocation must be carefully considered to maximise the efficacy of the procedure. Although the absolute efficacy is undebated - it may help HIV-positive patients with ESRD - the relative efficacy may not be as clear. If transplantation in an HIV-positive recipient versus an HIV-negative patient results in a lower quality of life and reduced patient survival, the organ could be preferentially allocated to the latter [5]. However, efficacy is not the only criterion determining organ allocation, and some argued in favour of transplantation even in pre-HAART era patients whose expected survival was less likely. One such argument was that medical urgency should be taken into account, as it is in the case of retransplantation [6] - patients routinely receive a second transplant despite the associated increase in mortality. Equity in access to organs should therefore ensure that HIV-positive patients receive the appropriate treatment when presenting with ESRD.

HAART is a combination drug therapy that consists of two NRTIs with one other class of antiretroviral drug - a PI, non-NRTI (NNRTI) or integrase strand transfer inhibitor (INSTI). Before its introduction in 1995, outcomes of cadaveric renal transplantation were significantly poorer in HIV seropositive patients than in those without HIV [7]. With the introduction of HAART, the premise that post-transplant treatment would exacerbate an already immunocompromised state was dismissed. The incidence of AIDS-defining illnesses fell compared to pre-existing therapy, as well as the incidence of AIDS-associated opportunistic infection [8]. A 2001 study on the outcomes of solid organ transplantation in HIV-positive recipients found that there was no progression of HIV, deterioration of CD4 count or development of AIDS-associated opportunistic infection observed with the immunosuppression regime given post-transplant [3]. In fact, rejection was a significant problem in renal transplant patients, with biopsy-proven rejection occurring in four out of six recipients, showing a capacity for post-transplant patients to mount an immune response. A follow-up study in 2003 reported that 10 out of 10 HIV-positive kidney transplant recipients were alive with functioning grafts after a mean 480-day follow-up period [9]. It is therefore not surprising that since the turn of the century, chronic diseases have replaced opportunistic infections as the leading cause of death among HIV-positive patients, including a rise in the incidence of HIV-associated nephropathy (HIVAN). The rising burden of ESRD on this population has sparked a reconsideration of HIV as a contraindication to kidney transplantation [5].

Pharmacological management of HIV-positive transplant recipients

The immunosuppressive regimen in kidney transplant recipients is similar in both HIV-positive and negative individuals, comprising induction and maintenance management [10], although in the former group it is combined with potent ART. Induction therapy is administered around the time of transplantation and is more immunosuppressive, with the aim of preventing acute allograft rejection (AR) and reducing the later need for maintenance drugs that are known to cause toxicity. It normally includes a biologic antibody - most commonly rabbit antithymocyte globulin (rATG) and basiliximab (interleukin-2 receptor antagonist) - and high-dose glucocorticoids. Maintenance is also started on the day of transplant in HIV-negative individuals and continues for the duration of the allograft. Higher risk groups, such as HIV-positive individuals or younger recipients, will often have test doses before transplantation to determine the appropriate post-transplant dosage [4]. This minimises the risk of suboptimal immunosuppression in the early post-transplant period. In most HIV patients (as with our patient), a triple-therapy approach is used comprising a calcineurin inhibitor (such as tacrolimus or cyclosporin), an antimetabolite (such as mycophenolate) and additional glucocorticoids. Other agents such as sirolimus (an mTOR inhibitor) are considered in patients who do not tolerate calcineurin inhibition, which carries a risk of graft nephrotoxicity [10].

Of relevance in the case of our patient is the use of the monoclonal antibody alemtuzumab as induction immunosuppression. Although unlicensed in the UK for transplant or oncological uses, it has been associated with reduced AR rates in low-risk HIV-negative recipients. In a study by Tan et al. [11], three HIV-positive patients received live donor renal allografts, with intraoperative alemtuzumab preconditioning followed by tacrolimus induction therapy. In all three of these recipients, there was no acute rejection, HIV viral loads remained undetectable and CD4 counts slowly recovered. In contrast, four recipients of deceased donor kidneys who did not receive intraoperative alemtuzumab showed higher rates of acute rejection (75%). The data from these studies suggest that alemtuzumab seems safe and effective in preventing graft rejection and justifies its inclusion in our patient’s post-transplant regimen. There is not much literature surrounding the use of alemtuzumab in this population, however, and it certainly warrants further investigation. In particular, the long-term effects of its extreme lymphocyte-depleting effect on HIV-positive recipients are yet to be elucidated.

HIV-positive recipients experience relatively high rates of acute AR. The largest prospective multicentre investigation of kidney transplantation in HAART patients, conducted in the US, found that AR occurred in 31% of patients at one year and 41% at three years - three to four times the reported figure in HIV-negative individuals [12]. These higher rates of rejection also suggest a benefit of the more aggressive induction antibody therapy. Indeed, patients receiving ATG did not demonstrate the same relative risk of AR and graft loss when compared to their HIV-negative counterparts and had a 2.6-fold lower risk of AR than HIV-positive patients who did not receive antibody induction [13]. Conversely, HIV-positive patients on sirolimus had an increased AR risk; the conclusion was to support a role for antibody induction and caution against mTOR inhibitor-based maintenance therapy in HIV-positive transplant recipients.

Although the rates of AR are reduced by antibody induction, the underlying immunosuppressed state of HIV-positive recipients is a concern when administering ATG or basiliximab. Patients may theoretically experience a prolonged lymphocyte depletion and a subsequent susceptibility to opportunistic infections. However, in a study of 830 HIV-positive kidney transplant recipients, neither induction agent was associated with an increased risk of infection when compared to no induction therapy, and patients on these drugs spent fewer days in hospital [14]. In fact, those receiving ATG (the most potent agent) had the lowest infection rates. Given that antibody induction does not convey a greater risk of infection, and additional prophylaxis against opportunistic pathogens such as cytomegalovirus, Pneumocystis jirovecii and Mycobacterium tuberculosis is prescribed as standard, antibody agents are strongly considered in such patients.

Although all HIV-positive patients must be on a stable HAART regimen to be considered for transplant, the cocktail of drugs they take may need modification before the procedure. Both NNRTIs and PIs carry an increased risk of interacting with immunosuppressive agents. PIs require dosing with boosting agents such as ritonavir, which are potent inhibitors of the cytochrome P450 system. Calcineurin inhibitors are a substrate for this system, and subsequently, PIs can reduce the metabolism of tacrolimus or cyclosporin and increase their blood levels - there are reported cases where tacrolimus dosage was reduced up to 120-fold when given alongside a ritonavir-boosted PI [15]. It is common practice, therefore, to give a trial dose before listing for transplant to determine the likely required dose following transplant. Glucocorticoids - another mainstay of post-transplant immunosuppression - are also metabolised by the cytochrome P450 system, and a reduction in glucocorticoid clearance has been reported in patients on PI-based HAART, leading to Cushing’s syndrome amongst other complications15. NNRTIs, conversely, are known inducers of cytochrome P450 and may reduce the calcineurin inhibitor blood concentration; this may necessitate a 1.5 to 2-fold increase in the calcineurin inhibitor dose [15].

With these risks in mind, many patients are taken off a PI or NNRTI-based regimen pre-transplant and switched to an INSTI. Cytochrome P450 isoforms constitute only a minor component of the metabolism of dolutegravir (a first-line INSTI), reducing the risk of drug interactions, which permits their co-administration with immunosuppressive drugs with minimal dose adjustment [16]. In the context of kidney transplantation, INSTIs have been shown to reduce the rate of AR when compared to non-INSTI-based regimens and meet some essential criteria for HAART regimens in organ transplant recipients; potency, lack of toxicity or interaction, and easy dosing. It would seem that, with some adjustment to their HAART routine and careful monitoring, outcomes for HIV-positive transplant recipients are heading towards those of their non-infected counterparts - and have been completely transformed since the pre-HAART era.

HIV-positive kidney donation

Although graft survival rates have been found to be similar in HIV-positive and HIV-negative patients when donated from an HIV-negative donor, there has been growing interest in whether a kidney could be successfully transplanted from a seropositive donor. The precedent for this approach was set by a group in South Africa in 2008 [17], where HIV was still an exclusion criterion for transplant and the availability of dialysis for ESRD patients was limited. Four transplantations were performed from two deceased donors who had no history of serious opportunistic infection or malignancy and had normal renal biopsies without evidence of proteinuria. Although the test group was small, all four patients had a good renal function at 12 months after transplantation without clinically significant graft rejection. A follow-up study of 27 transplantation cases reported 84% patient survival at both one and three years post-transplant, with rejection rates of 8% and 22% respectively [18]. HIV viral levels remained undetectable in the blood after transplantation. The study concluded that HIV-positive donation may be a viable way to increase the donor pool and reduce waiting times for patients with HIVAN, especially in countries where RRT cannot cope with the burden of HIV. A similar study in the UK followed, presenting a case report of two patients remaining in virological control, without episodes of rejection or opportunistic infection, following a donation from a deceased HIV-positive individual [19].

The caution in this approach stems from multiple risks. There is a potential for HIV disease progression to accelerate in the recipient, by superinfection with a different HIV clade or recombinant virus. Additionally, given the increased incidence of ESRD in the HIV-positive population, HIV donor nephrectomy was considered a higher risk than in uninfected donors. A study by the Segev group investigated the risk of ESRD in potential live donors and compared their estimates with similar clinical scenarios [20]. Their estimated risk increase in a 40-year-old HIV patient was comparable to the risk in HIV-negative cigarette smokers - which is not a contraindication to kidney donation. They also observed that the risk of ESRD in this population correlated directly with the quantity of replicating virus, which can be managed by HAART. In short, kidney donation from an HIV-positive donor (deceased or living) appears to be a real therapeutic option if donors are carefully selected, with a real but acceptable additional ESRD risk, and may significantly boost the availability of transplants for HIV patients with ESRD.

## Conclusions

The advent of HAART has dramatically changed the approach to transplantation in the HIV-positive recipient, increasing the eligibility of such patients for renal transplants from HIV-negative donors. It has also reduced the burden of opportunistic infection, allowing chronic diseases such as HIVAN to replace infection as the leading cause of death in HIV-positive patients, making access to renal transplants all the more important. The post-transplant pharmacological management in seropositive patients requires careful monitoring, but - as seen in the case of Mrs. X, who had virological control - the immunosuppressive regimen is largely the same, and similar rates of acute rejection compared to seronegative patients if immunosuppressive therapy is optimised. There is also growing potential for renal transplants from HIV-positive donors, with early studies demonstrating low rates of rejection and opportunistic infection following transplantation if donors are carefully selected. The therapeutic potential of HIV-positive donation warrants further study and presents an important pathway to increase access to RRT in HIV-positive patients with ESRD.
